# Hepatic steatosis secondary to capecitabine: a case report

**DOI:** 10.1186/1752-1947-4-227

**Published:** 2010-07-27

**Authors:** Sheray N Chin, Tae K Kim, Lillian L Siu

**Affiliations:** 1 Division of Medical Oncology and Hematology, Princess Margaret Hospital, University of Toronto, University Avenue, Suite 5-718, Toronto, ON M5G 2M9, Canada; 2 Department of Medical Imaging, Toronto General Hospital, University of Toronto, University Avenue, Toronto, Ontario M5G 2N2, Canada

## Abstract

**Introduction:**

There are no known case reports of hepatic steatosis caused by oral fluoropyrimidines such as capecitabine. With increasing use of capecitabine since its approval for the treatment of metastatic colon cancer in 2001, and more recently for adjuvant treatment of colon cancer and treatment of metastatic breast cancer, we can anticipate increased recognition of potential toxicities associated with this 5-fluorouracil derivative.

**Case presentation:**

We report the case of a 74-year-old Armenian woman who received capecitabine as adjuvant treatment for colon cancer and subsequently developed abnormal liver biochemical tests and radiographic findings in keeping with hepatic steatosis. There was complete reversal of liver enzyme abnormalities with discontinuation of the drug and this patient represents a case of reversible liver injury due to capecitabine.

**Conclusion:**

In this original case report, capecitabine use was associated with hepatic steatosis. It is important for clinicians to recognize and monitor for this potential toxicity, which may be a cause of abnormal liver enzymes in this patient population.

## Introduction

Capecitabine is an orally administered precursor of 5-fluorouracil (5-FU), a fluoropyrimidine antimetabolite. It is converted to 5-FU preferentially in tumor tissue, and also in the liver, by way of a three-step enzymatic cascade [1]. Capecitabine is a relatively new agent, with FDA approval in 2001 for use as an alternative to the Mayo Clinic 5-FU/folinic acid regimen for metastatic colon cancer. It has since been approved for use in the adjuvant treatment of colon cancer, as well as for metastatic breast cancer.

Hepatic steatosis, a mild manifestation of non-alcoholic fatty liver disease (NAFLD), may occur after treatment with 5-FU. This has become a more recognized complication in the era of hepatic surgery for colorectal liver metastases, where hepatic steatosis is associated with increased post-operative morbidity [2]. Peppercorn et al. [3] found that 47% of patients with colorectal liver metastases treated with systemic 5-FU and folinic acid had computed tomography (CT) findings consistent with fatty change. Another report described laboratory abnormalities consistent with hepatic toxicity in 40% of patients who received adjuvant therapy with 5-FU and levamisole after undergoing surgical resection for Stage II or III colon cancer, with CT and biopsy evidence of steatosis in a few cases [4]. There are, however, no known reports of liver damage caused by oral fluoropyrimidines [5].

## Case presentation

A 74-year-old Armenian woman with Stage III colon cancer was treated in the adjuvant setting with capecitabine. Comorbid conditions included type II diabetes mellitus, hypertension and gastroesophageal reflux disease; her concomitant medications included glyburide, metformin, telmisartan, atenolol and lansoprazole. She had no known hepatic disease, no history suggestive of Hepatitis B or C exposure and did not drink alcohol. Baseline liver enzymes and bilirubin were normal (AST 11 U/L [normal < 35 U/L], ALT 7 U/L [normal < 40 U/L], bilirubin 7 μmol/L [normal < 22 μmol/L]) and pre-treatment staging investigations including magnetic resonance imaging (MRI) of liver (performed due to CT contrast allergy) demonstrated no evidence of metastatic disease. Adjuvant chemotherapy was initiated with capecitabine at 1000 mg/m^2 ^twice daily for 14 days every three weeks, for a planned total of eight cycles. She developed Grade 2 diarrhea with the first cycle, with dose reduction to 750 mg/m^2 ^twice daily, which was well tolerated.

Her transaminases started to rise after the third cycle of capecitabine (AST 44 U/L, ALT 57 U/L, bilirubin 18 μmol/L), with further elevation as well as mildly increased bilirubin after the fourth cycle (AST 73 U/L, ALT 101 U/L, bilirubin 24 μmol/L). She remained anicteric and had no symptoms of hepatic dysfunction. Capecitabine was delayed while imaging investigations were arranged to rule out the possibility of hepatic dysfunction due to liver metastases. Other causes of liver disease such as viral hepatitis were considered and ruled out with negative serology for Hepatitis B and C.

**Figure 1:**
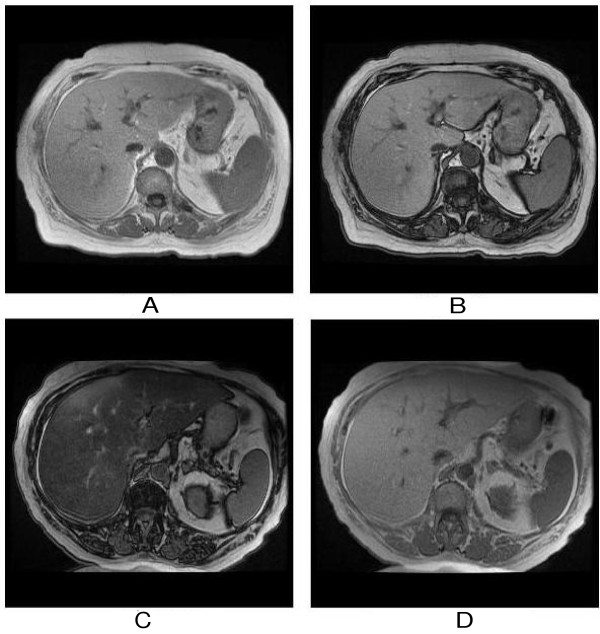
**Capecitabine-induced hepatic steatosis in a 74-year-old woman**. Dual-echo, chemical shift gradient-echo T1-weighted magnetic resonance images show no evidence of hepatic steatosis which is demonstrated by the signal intensity of the liver on the in-phase (TR/TE, 150/4.5) image (A) similar to that of the out-of-phase (150/2.3) image (B). Three-month follow-up magnetic resonance images after treatment with capecitabine clearly show newly developed severe hepatic steatosis which is seen as a drop of the signal intensity of the liver on the out-of-phase (150/2.3) image (C) compared with the in-phase (150/4.5) image (D).

MRI of her abdomen demonstrated marked hepatomegaly with severe fatty infiltration of the liver; the significant signal drop in out-of-phase compared to in-phase imaging, which was not present on baseline scans, confirmed severe hepatic steatosis (see Figure 1A-D). There was no evidence of metastatic disease.

A clinical diagnosis of capecitabine-induced hepatic steatosis was made. Capecitabine was held and after one month, there was a decline in the transaminases and bilirubin to normal (AST 27 U/L, ALT 35 U/L, bilirubin 13 μmol/L). After consultation with the hepatology service, it was decided that the risk of disease recurrence likely outweighed the risk of irreversible liver injury due to capecitabine. After discussion with the patient and her family, it was decided to cautiously rechallenge her, with close monitoring of the liver enzymes. Transaminases and bilirubin remain normal after the first rechallenge cycle (AST 30 U/L, ALT 41 U/L, bilirubin 9 μmol/L).

## Discussion

The spectrum of liver changes associated with fat accumulation in hepatocytes is termed non-alcoholic fatty liver disease (NAFLD) [2]. Although abnormal liver function tests and radiographic findings may be suggestive of NAFLD, histological evaluation remains the only way to assess hepatocyte damage and to distinguish 'simple' steatosis from steatosis with inflammation, or the more serious steatohepatitis, which can progress to cirrhosis [6].

The classic findings of fatty liver associated with abnormal liver tests and the improvement in transaminases upon drug interruption render NAFLD due to capecitabine the most likely diagnosis in our patient. A liver biopsy for histological confirmation was not pursued in our patient, who fortunately had complete reversal of liver enzyme abnormalities with discontinuation of the drug, and therefore likely had a reversible mild form of steatosis.

## Conclusion

In this original case report, the patient presented represents a case of hepatic steatosis associated with the use of capecitabine. It is important for clinicians to recognize and monitor for this potential toxicity, which may be a cause of hepatic dysfunction in this patient population. This is especially important as these patients are also at risk for hepatic metastases, which may present similarly and need to be considered in the differential diagnosis. With increased capecitabine use, we anticipate more cases of NAFLD associated with this 5-FU derivative.

## Consent

Written informed consent was obtained from the patient for publication of this case report and any accompanying images. A copy of the written consent is available for review by the Editor-in-Chief of this journal.

## Competing interests

The authors declare that they have no competing interests.

## Authors' contributions

LS made the initial clinical diagnosis, with the assistance of TK who interpreted the radiological findings regarding hepatic steatosis. SC performed the literature review and was responsible for writing the manuscript. All authors read and approved the final manuscript.

## References

[B1] Miwa M Ura M Nishada M Sawada N Ishikawa T Mori K Shimma N Umeda I Ishitsuka H Design of a novel oral fluoropyrimidine carbamate, capecitabine, which generates 5-fluorouracil selectively in tumors by enzymes concentrated in human liver and cancer tissue Eur J Cancer 1998 34 1274 1281 10.1016/S0959-8049(98)00058-6 9849491

[B2] Zorzi D Laurent A Pawlik TM Vauthey J-N Abdalla EK Chemotherapy-associated hepatotoxicity and surgery for colorectal metastases Br J Surg 2007 94 274 286 10.1002/bjs.5719 17315288

[B3] Peppercorn PD Rezneck RH Wilson P Slevin ML Gupta RK Demonstration of hepatic steatosis by computerized tomography in patients receiving 5-Fluorouracil based chemotherapy for advanced colorectal cancer Br J Cancer 1998 77 2008 2011 9667683 10.1038/bjc.1998.333 PMC2150330

[B4] Moertel CG Fleming TR Macdonald JS Haller DG Laurie JA Hepatic toxicity associated with fluorauracil plus levamisole adjuvant therapy J Clin Oncol 1993 11 2386 2390 8246027 10.1200/JCO.1993.11.12.2386

[B5] King PD Perry MC Hepatotoxicity of chemotherapy Oncologist 2001 6 162 176 10.1634/theoncologist.6-2-162 11306728

[B6] Brunt EM Nonalcoholic steatohepatitis Semin Liver Dis 2004 24 3 20 15085483 10.1055/s-2004-823098

